# Achieving Flow: An Exploratory Investigation of Elite College Athletes and Musicians

**DOI:** 10.3389/fpsyg.2022.831508

**Published:** 2022-03-30

**Authors:** Roberta Antonini Philippe, Sarah Morgana Singer, Joshua E. E. Jaeger, Michele Biasutti, Scott Sinnett

**Affiliations:** ^1^Institute of Sport Sciences, SSP, University of Lausanne, Lausanne, Switzerland; ^2^Department of Psychology, University of Bern, Bern, Switzerland; ^3^FISPPA Department, University of Padua, Padua, Italy; ^4^Department of Psychology, University of Hawaii at Mānoa, Honolulu, HI, United States

**Keywords:** flow experience, qualitative study, elicitation interview, dynamics of flow, role of emotions

## Abstract

While studies on the characteristics of flow states and their relation to peak performance exist, little is known about the dynamics by which flow states emerge and develop over time. The current paper qualitatively explores the necessary pre-conditions to enter flow, and the development of flow over time until its termination. Using an elicitation interview, participants (10 athletes and 12 musicians) were asked to recall their flow experiences in sports or music performances. The analysis resulted in the identification of the following three phases that athletes and musicians experience during flow: (1) Preparation to enter flow; (2) Entry into the flow state and; (3) Exit from the flow state. These three phases are characterized by several sub-themes contributing to the experience of flow. The function of emotions is crucial, as they play a core role across all three phases and regulate flow over time. The findings provide insights into the phenomenological characteristics of the transition and maintenance of the three proposed phases and the temporal dynamics of flow.

## Introduction

Athletes, artists, and musicians all aspire to enter and maintain a state of flow when performing or competing. When experiencing flow, performers feel at their best and often reach their highest level (Palomäki et al., 2021). Flow state theory is grounded in the field of positive psychology and originated with Csikszentmihalyi (1990), the founder of the concept, who defined flow as a particular state of optimal activation in which participants are completely immersed in their activity (Csikszentmihalyi, 1975; Demontrond and Gaudreau, 2008). Flow is multidimensional and is felt when performers could be described as self-sufficient (Demontrond and Gaudreau, 2008) while engaging in an activity that is important for them (Csikszentmihalyi, 1990). Performers describe flow as becoming at “one” with the activity leading them to enter another reality in which they are entirely absorbed by what is being undertaken (MacDonald et al., 2006).

Flow is composed of cognitive, physiological, and affective factors (Habe et al., 2019), and is characterized by a balance between the perceived challenges or opportunities for action that stretch but do not overmatch existing skills, and clear goals and immediate feedback about the progress being made (Csikszentmihalyi and Nakamura, 2002). The individual who experiences flow feels great joy and happiness and aims to relive this experience (Csikszentmihalyi and Bouffard, 2017). According to Csikszentmihalyi (1990), the experience of flow can be characterized by the following nine dimensions: (1) a challenge–skills balance to feel engaged but not overwhelmed; (2) clear objectives allowing one to concentrate on the current task and knowing what is coming next, while reducing distractions and stress; (3) an action-awareness merging, meaning the person is completely absorbed by the situation; (4) clear and unambiguous feedback, implying that they know what they are doing at all times when experiencing flow; (5) a high, task-related, concentration that absorbs the person in the activity without being distracted by unrelated things; (6) an absolute sense of control; (7) a loss of self-consciousness, leading to the person being so immersed in the activity that there is a lack of ego-oriented protection; (8) the transformation of time, meaning that the time either slows down or flies by when experiencing flow; and, finally (9) an autotelic experience, as the activity becomes an end in itself. Experiencing these dimensions lead performers to feel totally immersed in the activity and have the feeling that no effort is required (Harris et al., 2017).

Some scholars have criticized the concept of flow for its vagueness (see Harris et al., 2017). However, the reshaped multidimensional description of flow, reported above within the framework of the nine dimensions, presents flow as a less frequent and more specific event, which closely aligns with the reality of the phenomenon (Csikszentmihalyi and Bouffard, 2017), and arguably is more easily measured and assessed.

### Flow Within Athletes and Musicians

The concept of flow has attracted researchers from different areas and has been studied across a wide range of fields, such as work and leisure (Csikszentmihalyi and LeFevre, 1989), teaching (Diaz and Silveira, 2012), dance (Biasutti and Habe, 2021), theatre (Martin and Cutler, 2002), music (MacDonald et al., 2006), and athletics (Kimiecik and Jackson, 2002; Sinnett et al., 2020). It has been shown that the experience of flow and performance are positively related across activities.

In sports, the flow state has a strong positive relationship with the level of performance (Csikszentmihalyi and Nakamura, 2002). In addition to being linked with high-quality performance, flow states are associated with confidence, the ease to which sport-specific actions are executed, and automaticity (Harris et al., 2017). Flow, characterized as a state of optimal functioning (Asakawa, 2004), allows a person to transcend one’s average and usual skill level to fully realize one’s potential. Flow also impacts the acquisition of new skills (Csikszentmihalyi and LeFevre, 1989) and the improvement of skills (Massimini and Carli, 1988). As a challenging activity is mastered, it becomes less involving, and the challenge it represents becomes simpler. To continue to experience a flow state, people must engage in more complex challenges, implying the development and improvement of their skills. Flow has been widely studied in a sports context (Swann et al., 2015), with increased focus given to better understanding the specificities of this experience within this context of performance (Chavez, 2008).

Emotions seems have a core role in determining flow (Rodgers and Tajet-Foxell, 2011). A model called the individual zones of optimal functioning (IZOF) was developed by Hanin (2000) as a reference for qualitative and quantitative examinations of the framework and function of emotional experiences related to successful or poor performances. A multidimensional description of performance-related psychobiosocial states, focusing on IZOF-based predictions of emotion–performance relationships, suggests tentative functional interpretations of emotion–performance relationships with an emphasis on their bi-directionality and interaction effects.

Progressively, other domains have recently been interested in the context of flow, particularly in artistic fields, such as music. In music, flow was mainly described in improvisation, interpretation, and composition, as musicians are often immersed in these activities. Biasutti (2017a, p. 5) explains that “[musicians] feel completely absorbed through a strong sense of identification with the music”. Flow is considered to be a common experience for musicians and scholars highlighted that flow states are experienced quite frequently by music students (Sinnamon et al., 2012), and that the majority of professional classical orchestral musicians regularly experience flow (Cohen and Bodner, 2019). However, unlike research in sports or occupational psychology, fewer studies have been devoted to the study of flow in the field of music (Chirico et al., 2015). Researchers agree that it is an area in need of further investigation, given the benefits of achieving such a state, both for performance and for the way it is experienced (Wrigley and Emmerson, 2011).

Music research examined aspects related to the factors that contribute to flow experiences, the transmission and group experience of flow, the association of flow with a range of positive outcomes, and the psychophysiological aspects of flow (Tan and Sin, 2021). In addition, topics covered in flow studies have included self-regulated behaviors and job resources. In classically trained musicians, flow was found to be a function of self-regulated behaviors, such as personal resources, with practice organization and self-regulation contributing to flow experience with indicators related to musical skills, tasks, the clarity of goals and feedback, concentration and control over the activities (Araújo and Hein, 2016). Regarding music teachers and students, aspects of job resources, including performance feedback, autonomy, social support, and supervisory coaching all have a positive influence on the balance between teachers’ challenges and skills and contribute to the experience of flow (Bakker, 2005).

Experiencing flow has many beneficial consequences for performers’ wellbeing. Flow state is associated with a positive experience and leads to great pleasure in carrying out the activity and its associated feeling of fulfillment (Csikszentmihalyi, 1990). As Csikszentmihalyi (1990, p. 6) argued, “if a person sets out to achieve a difficult enough goal, from which all other goals logically follow and if he or she invests all the energy in developing skills to reach that goal, then actions and feelings will be in harmony and the separate parts of life will fit together and each activity will make sense in the present, as well as in view of the past and the future.” Therefore, an important challenge is to understand the parameters that lead to the attainment of flow (Csikszentmihalyi, 2004).

### Challenges to Achieving Flow

According to Csikszentmihalyi (1999), flow is not a phenomenon that can be consciously triggered by a person’s will. With that said, there are factors that can facilitate its emergence (Jackson and Csikszentmihalyi, 1999). Accordingly, flow states would be more easily accessible if the necessary conditions to enter flow are facilitated (Csikszentmihalyi, 1999). We can wonder if it is possible to achieve flow at all stages of learning. This leads to an important question: what exactly are the conditions that lead to a higher probability of achieving flow?

Recent research has shown that appropriate practice generates a certain control over the emergence of flow in athletes or musicians (Jackson and Csikszentmihalyi, 1999). Factors such as being focused and convinced that nothing is more important than the experience in question, having the right skills for the demands of the task, and being able to direct one’s attention to the task at hand during the activity (Biasutti, 2017a) are some conditions that must be fulfilled to experience flow. Biasutti (2017a) also suggests that having clear mental plans for the performance and knowing all the variables in advance can help the performer attain a flow state. Kirchner (2011) posited that there can be a tendency to experience flow if certain predispositions are met that enable flow to emerge. He identified five constitutive dispositions of this tendency to experience a flow state: self-confidence, the desire to experiment and express something through the activity, having goals, the ability to maintain one’s attention, and the ability to perform without self-criticism.

Flow can be experienced at several levels, with the highest form occurring during the activity itself. The normal concept of flow is considered as a state characterized by a natural and regular “challenge–skills balance” experience allowing performers to be absorbed in their experience, but to still be able to have some kind of “action-awareness.” The ultimate experience of flow could include a transcendental experience in which performers are often described as being in a trance-like state.

While experiencing flow, performers have an increased in skills and achieve their maximum potential. This ascent, whether technical, personal, or mental, is influenced by the situation. Flow states are experienced during judged performances (e.g., concerts and competitions), which can act as flow facilitators (Biasutti, 2017a). On the other hand, an unfavorable environment, as well as internal factors like anxiety and impatience all have the ability to hinder the emergence of flow. Flow states and the music performance anxiety (MPA) could be considered antithetical experiences (Cohen, and Bodner, 2019), and pre-performance anxiety could play a role in the achievement of optimal performance states (Hanin, 2000; Kenny and Osborne, 2006; Rodgers and Tajet-Foxell, 2011).

Several studies (see for example, Swann et al., 2016, 2017) have led to a better understanding of how states of flow emerge and occur. Flow is considered a rare and elusive state (Swann et al., 2015), with most knowledge based on factors simply associated with its occurrence (e.g., optimal environmental conditions) rather than causal mechanisms (Aherne et al., 2011). Recent studies have explored the chronology of the onset of flow, suggesting that flow occurs in exploratory contexts involving novelty, discovery, uncertainty, or experimentation (Swann et al., 2016, 2017). In such contexts, flow emerges as a result of a gradual accumulation of confidence during performance (Swann et al., 2016, 2017). Specifically, a constructive first event, leading to positive feedback increases the performer’s confidence. This process is repeated until the performer reaches a level of total confidence that allows him/her to pursue the objectives, and challenge him/herself to explore his/her own limits and thus enter into a flow state.

Another important issue for our understanding of flow relates to the extent to which flow states can be controlled and how the frequency and intensity of flow experiences can be systematically increased. The literature is currently divided on this issue, with some scholars arguing that with appropriate exercises, athletes and musicians can develop control over flow states (Jackson, 1995), while others consider that it is difficult to regulate flow, although it is possible to acquire a mental attitude that facilitates reaching flow (Csikszentmihalyi, 1990). For example, in a study involving elite athletes, Jackson (1995) collected views about the controllability of flow in which 71% of the athletes considered flow to be under volitional control through elements, such as physical and mental preparation. Csikszentmihalyi (1990) argued that flow states occur when participants are convinced that nothing is more important than the activity at hand and that they have demonstrated the ability to master the situation. Expert musicians reference several steps when preparing themselves for peak performance, with regard to the following three areas: cognitions, emotions, and behaviors (i.e., thoughts, feelings, and activities). Activities, such as managing emotions, managing thoughts, developing intrinsic motivation, concentrating and focus, mental practice, imagery and visualization, calming the body and mind, and pre-performance plans, could be undertaken in order to facilitate flow appearance (Sinnamon, 2020).

While numerous studies have explored flow states in sports and music, the characteristics and actions that lead to the entrance and/or exit from flow states are difficult to clearly identify and are largely unknown. Moreover, it is important for flow research to ascertain detailed aspects and to be specific about the possible directions of the study. It is relevant not only to aid rigorous research methods but also to define useful routes for musicians and educators that could orient on how to achieve flow and if there are similarities, differences, and implications within the field of music performance.

The aim of this study is to understand the dynamics by which athletes and musicians specifically enter and exit the flow state. The dynamic that unfolds regarding performance could be understood considering the specificity of the interaction between the situation and the individual (i.e., his/her actions). A qualitative approach was considered the best method to examine the pre-conditions to enter flow and the development of flow over time until its termination.

## Materials and Methods

### Participants

Twenty-two students from Hawaii University took part in this study, including ten athletes and twelve musicians aged between 19 and 31 years old (*M* = 22.7; *SD* = 3.9; 10 female and 12 male).

The participants were recruited during an announcement at practice sessions and also by word of mouth. To obtain a general picture of the flow experience, the population included different types of expertise in athletes (one climber, two runners, and seven tennis players) and in musicians (one piano, two flutes, two saxophones, one basson, one clarinet, one trumpet, one oboe, one trombone, one tuba, and one percussion). The participants were recruited from local universities in the Honolulu area (athletes) or directly from the music program at the University of Hawaii at Mānoa. All participants compete or perform at exceptional levels (e.g., NCAA Division II tennis players; performing musicians). The athletes had between 3 and 20 (*M =* 13; SD = 5.94) years of experience and practiced between 5 and 25 (*M =* 15; SD = 7.18) hours per week on average. The musicians had between 3 and 17 (*M =* 8.6; SD = 3.22) years of experience and practiced between 2 and 22.5 (*M* = 8.3; SD = 6.23) hours per week on average. As previous research has shown that skill level is correlated with the experience of flow (Catley and Duda, 1997; Engeser and Rheinberg, 2008), expertise was operationally defined as regular practice over several years in a particular discipline. All participants provided written informed consent prior to the start of the study. As compensation, participants were offered the opportunity to attend a mental preparation seminar conducted by one of the authors (RAP). The present study was reviewed and approved by Committee on Human Subjects at the University of Hawaii at Manoa.

### Procedure and Methods

Athletes and musicians were contacted *via* e-mail (that was provided during the study announcements), offering them the opportunity to voluntarily participate in this study. Participants were asked if they had ever heard of the concept of flow. In order to provide participants with a uniform understanding of flow, they were first given a definition of this concept. Specifically, participants were asked if they had ever experienced a flow state in accordance with Csikszentmihalyi (1990) who defined flow as a mental state of operation in which a person performing an activity is fully immersed in a feeling of energized focus, full involvement, and enjoyment in the process of the activity. As such, the flow experiences that were discussed were generated by each participant’s recollection of a relevant and personally experienced performance (athletic or music related) where they experienced flow. Elicitation interviews were carried out individually and conducted to collect two forms of data: (a) athletes and musicians were asked to draw a curve representing the evolution of the flow state they had experienced (for an example see, Supplementary Figure 1) and (b) the interviews were recorded and then transcribed to analyze their content.

- The representation of the temporal evolution of the participants’ flow states in the form of a graph allows a better understanding of their subjective experience. Drawing the graphs makes it possible to transform the flow experience into something tangible that can be referred to during the interview. The graph can therefore help the participant to remember the desired moment and to relive it (Drasch and Matthes, 2013). The interviewer asked the participant to illustrate the temporality of a past situation where he or she had experienced the flow state through the discussion of the graph. The illustration facilitated the identification of different phases that an athlete or musician experienced before or after reaching the flow state. The goal was to identify the affects/emotions, thoughts, and actions that characterized each phases. The collection of the data was therefore conducted through elicitation interviews that provide access to past experiences. Elicitation interviewing has been used in other studies in the field of sport psychology by various scholars in the field of sport psychology who have examined the temporal development of individual and collective experiences (Antonini Philippe et al., 2016; Rochat et al., 2017). Analogous methodological approaches have been used in the artistic field in research on contemporary musical composition (Donin and Theureau, 2008) and in a study looking at the preparation of musicians before performing in a contest (Antonini Philippe and Güsewell, 2016).
- Athletes and musicians took part in elicitation interviews to highlight and explore the elements that facilitate the emergence and disappearance of the flow state. The elicitation interviews (Theureau, 2010) lasted on average 30 min and allowed participants to share their experiences of flow. The use of this interview method provides verbalized data about a past experience. The interview method could be used to extract non-verbal data, such as facial expressions or gestures, although these types of measures were not used in this study.

The interview was based on the specific situation chosen and graphically represented by the athlete or musician during which he or she recalled experiencing a flow state. The participant was encouraged to recall a situation where they directly remembered being in a flow state and were asked to recall this situation in as detailed a manner as possible. The interviewer’s questions then focused on the temporality of this experience as well as on the thoughts, emotions, body sensations, and actions of the participant. For example, the questions asked were as follows: How were your body sensations at the beginning of your performance? What did you think at this moment? What was the difference between these two moments? How did you feel physically and mentally? What were you focusing on? Can you describe your perception of time at this moment? What strategies did you put in place? Although the questions asked targeted these aspects, no interview guide was developed or used during the interviews.

To enable the transcription and analysis of the collected data, the interviews were all recorded using an iPad. The audio files were then transcribed and anonymized. For ethical reasons and to ensure the anonymity of the participants, each participant received and signed an information sheet and a consent form allowing the analysis of the collected data. All participants had the right to not answer any of the questions or to interrupt the interview at any time if he or she wished to do so. The analysis of the corpora obtained during the interviews made it possible to put forward two phases preceding the flow state as well as one phase coming afterwards. Three of the authors of this article conducted the interviews due to the large number of participants.

### Data Analyses

The analysis procedure was data-driven rather than theory-driven (Charmaz, 2003) and was carried out using an inductive thematic approach, based on several steps (Braun and Clarke, 2006; Braun et al., 2016, see also Condon and Ogston, 1967 for seminal work on behavioral segmentation). First, the researchers reread the interviews several times to allow them to become familiar with their content. The analysis method requires that the relevant corpora be separated into meaningful units, which should be understood as unique ideas related to the research question. Once all meaningful units had been identified and named, the entirety of the interviews was reread to confirm the relevance of these units. The units that expressed similar ideas were then grouped under different sub-themes and categories, thus characterizing a significant phase in the evolution of the flow state. This entire step was done by hand; no software or automated help was used.

To guarantee a reliable analysis of the collected data, the themes and their contents were discussed within the research team (Smith and McGannon, 2018). When disagreements occurred during the categorization of the meaningful units, the researchers discussed them finding an agreement. To ensure that the researchers did not over-interpret the words of the participants, the results of the analysis are supported by verbatims from the interviews, which can be read in the following section. To guarantee anonymity, all participants were assigned the letter A (for the 10 athletes) and M (for the 12 musicians) and a different progressive number (i.e., A 1–10; M 1–12).

## Results

As a result of the analysis carried out, several themes and sub-themes emerged. Specifically, the analysis allowed the identification of two distinct phases which athletes and musicians go through before reaching the flow state: Phase 1: Preparation; Phase 2: Entry into the flow state. In addition, the analysis made it possible to identify a phase following the flow state: Phase 3: Flow state exit. In the section reported below, the three phases and their sub-themes are discussed (Table 1).

**Table 1:** The three phases and their sub-themes.

Preparation	Flow state entry	Flow state exit
(a) Warm-up	(a) Goal setting	(a) End of the performance
(b) Focus	(b) High level of involvement	(b) Physical experience
(c) Body feelings	(c) Control over performance	(c) Mental experience
(d) Emotional state	(d) Positive emotions and sensations	

### Phase 1: Preparation

Participants identified “preparation” as the first phase, which is composed of the following four sub-themes: (a) warm-up; (b) focus; (c) body feelings, and (d) emotional state.

#### Warm-Up

The warm-up is the phase in which performers prepare themselves for the activity. The warm-up could be both physical and mental. The physical warm-up aims to activate the body to be able to engage in the activity. For athletes, it may consist of small games or tasks that are less demanding than the final activity/performance. Musicians tune their instruments (e.g., for the string instruments) during this phase and test the instruments doing exercises, such as long tones and scales. They then play a few notes alone or with their ensemble.

« […] *I was just warming up and I was doing easier climbs and so, you know, there wasn’t play challenging. You know, I was making sure my hands are good and everything.* » (A1).

*« Jumping on the spot, little runs on the long distance, finally everything that allows me to wake up physically. » (A1).*

During the mental warm-up, participants try to visualize what they will have to do once they are engaged in their activity/performance. Some participants highlight that they say things to themselves to encourage and reassure themselves before performing. Musicians perform some relaxation exercises to control their heart rate and breathing.

« *So, I do a series of breathing exercises to control my heart rate and also control my breathing so that I can play comfortably without having to gasp for air* […] » (M12).

*« I tell myself that I can do it and that I’m ready, then I follow it up with cardiac coherence, which helps me a lot. » (M11).*

#### Focus

During the preparation phase, the level of focus of the participants varies and can be either internal or external to them. The internal focus consists of attention directed toward the body, sensations, or emotions as reported by one participant:

« […] *so I’m usually maybe focused on just get my legs moving enough to focus on how I’m feeling mentally or like any feedback that my legs would get for me because even if they are feeling sore or tired or something usually it gets a little better later on so I do not read too much into it then so more focus on just getting like a get moving and get a nice warm up*. » (A3).

*« When I feel tired, I focus on myself, the symptoms subside and the diffuculty decreases, as well as the fear, this fear that stops me in action.* » (A5).

Athletes report internal focus more than musicians do. Conversely, the external focus consists of a specific attention directed toward what is outside the musician or athlete, such as the audience as reported in this statement:

« *I was more like concerned about what other people were thinking of me and more distracted like I would check my phone more often* » (M12).

*« It’s all about concentration and I know that, but I cannot go back on myself. »* (M10).

Musicians report external focus more than athletes do. In general, internal focus is helpful for seeking concentration and is based on internal psychophysical resources, while external focus could have a negative impact and distract the performers.

#### Body Feelings

During the warm-up, participants’ body sensations can be either positive or negative. These sensations are often related to whether the body is tired or fresh or whether it is tense or relaxed. However, the purpose of the warm-up is to ensure that at the end of it, the body’s sensations are positive.

« […] *so, the body’s a little bit slower* […] *Usually that very beginning of the run just because I’m going from like a state of like sitting or something like that to get my body actually moving it’s a little it would probably be like in most cases the worst part of my run*. » (A3).

« *When I was going into here, so I felt kind of like tired and sluggish.* » (M11).

#### Emotional State

The final sub-theme reflects the emotional states shared by the participants. In the majority of cases, they reported experiencing negative emotions, such as anxiety or nervousness before performing. They then set up their personal routines to cope with the emotions they felt.

«*I think the closer I got to being on stage the more nervous I became so I was really trying to control my breathing and trying to like tell myself okay we can do this I just have to make sure we focus.*» (M5).

*«It’s a strange feeling, which I do not really like. Is it fear? I will not know how to define it, but it is unpleasant. »* (M5).

Musicians explained that when they arrived at the performance site, they prepare their instrument and check that it is in tune before playing a few notes alone or in groups. Their focus is more external: the musician’s attention can therefore be directed toward the audience or his or her external environment in general. During the preparation phase, the musicians do breathing exercises to control their respiration and heart rates. These exercises can be done at two different times: before training or just before going on stage.

Athletes begin their preparation with a physical warm-up to activate their bodies. The duration of the warm-up should depend on their body sensations, which is likely the reason for why their focus is mainly directed internally. Mental preparation can take place before, during, and/or after the body warm-up, depending on the athlete’s routines. The negative emotions felt by most participants grow with the arrival of the performance.

### Phase 2: Flow State Entry

Once their warm-up and preparation were completed, the participants enter a second phase which can be defined as a phase of evolution toward the flow state. This phase is composed of the following four sub-themes: (a) goal setting: (b) high level of involvement in the activity; (c) control over performance and (d) positive emotions and sensations.

#### Goal Setting

Performance is often associated with goals to be achieved. These goals can be either qualitative or quantitative. Quantitative goals are more commonly reported by athletes than musicians and, for example, could be a time or a score to reach. However, this depends on the sport being practiced. Qualitative goals are more likely to be reported by musicians and, for example, could be being tuned with the ensemble during the performance.

« […] *so I’m trying to keep my heart rate usually below 150 beats per minute and I’m kind of watching my time with that and I was able to meet the times I wanted while keeping my heart rate where it was I wasn’t seeing any like big jump in performance or anything like that but it’s kind of right on pace of what I expected and then after that did a full workout and basically hit the numbers I was expecting to so* » (A3).

« *We wanted to do well we want to do well wanted to represent our school well* » (M5).

#### High Level of Involvement in the Activity

Once participants are engaged in their performance, they describe that their level of concentration increases and that their attention is mostly directed to the task they are performing.

« *Because when it’s more dangerous, I have to become more concentrate, you know. If my holds are good, if my feet are good. But I do not have time to concentrate on that small pain* […] *so you really just gotta start to ignore everything else. It’s kind like when you are meditating. When you start to meditate you have all these thoughts in your head and as you are building up to that, when you are kind of in that proper good climb, al thoughts washes away*. […] *I think that’s one of the reasons I love climbing so much it really helps me just concentrating and ignore all the bothering things in your mind*. » (A3).

« *I guess more focus more involved in the activity in the sense that my mind is completely there* […] *it’s easier to focus it’s easier to gain attention* […] *as you rehearse more and more and more and more the focus level only goes up the attention only goes up because the distractions from things that happened 30 min or an hour ago no longer is relevant in your mind* […] » (M7).

#### Control Over Performance

During this second phase, the participants exercise some form of control over their performance despite the various challenges that may emerge from it or from their environment. In the case of athletes, they can apply certain tactics seen in training or plan the next steps of their performance. In the presence of challenges, they can adapt physically and mentally to them.

« […] *you try to concentrate on certain things like for example doing some returns or just put the ball in or you try to think of a tactic you want to do so it’s like you actually have a plan* […] » (A8).

« *It was more like what, is it somewhat challenging, can I figure out the problem and can I figure out which way to move my body, which holds need to be held in a certain order for me to figure it out and can I make the physical movement, mentally and physically* » (A1).

Musicians exercise some control over the music they produce by playing the right notes. They are also able to adapt to the conductor and their ensemble.

« […] *we go start going up we are already progressing through the music we are you know how we started there’s a high point coming up or and we can achieve that that high point in an efficient manner both playing as an individual and as an ensemble*» (M6).

#### Positive Emotions and Sensations

The second phase is also associated with positive body sensations as well as positive emotions. According to the participants, these positive emotions are correlated with the successful completion of their performance. In addition, they feel more energetic and confident.

« […] *and then by the time you actually saw me climbing that one route, that was pretty, I was pretty much in it. I was so, I was really enjoying the route*. […] » (A1).

« […] *I was a lot more energizing* […] *I felt a lot more awakened*» (M7).

The performance of athletes or musicians was associated with a goal that was set before they performed. In the first phase, the focus of the participants could vary: the musicians’ attention was directed externally, while the athletes’ attention was directed internally. Conversely, in this second phase, and once the performance had started, the attention of athletes and musicians was directed toward the task at hand. During their performance, participants had the ability to adapt to their environment. In the case of sports, athletes considered aspects, such as their opponent(s), the weather conditions, or the terrain. In the case of music, the musician considered the conductor or members of the ensemble as an example. The successful completion of the performance is linked to the positive emotions felt by both the athletes and musicians.

### Phase 3: Flow State Exit

According to the participants, once the flow state had been experienced, it gradually faded away. Various elements were identified as playing a major role in this exit from the flow state, which were characterized by the following three sub-themes: (a) End of the performance, (b) Physical experience, and (c) Mental experience.

#### End of the Performance

The termination of the flow state was associated with the end of something. Based on the experience shared by musicians, the flow state disappeared once a highlight of a piece was reached or once the performance had ended and the conductor was finished. In the case of athletes, the end of the flow state was also associated with the end of the performance but could also be due to a high number of errors committed or a decrease in level—participants associated the end of the flow state with a return to reality.

« *If it is the end of that piece yeah conductor stops there’s a few seconds of silence before the clapping starts and usually when that clapping starts is kind of went okay now we are back*. » (M10).

« *Then I moved closer to you guys I did a couple routes over there, and there are a couple of times where my foot slipped off. Well, really just one time that my foot just got slipped off, I wasn’t in sake of hurting myself and I did not worry or anything, but it certainly kind of brings you back to reality*. » (A1).

#### Physical Experience

This return to reality impacts the body experience; for example, athletes and musicians both reported physical fatigue due to effort. The sensation felt at that moment could diverge depending on the reason why the flow state had been interrupted. Thus, the end of the flow state linked to the termination of the performance was associated with sensations of relaxation, while the exit from the flow state linked to errors or a decrease in the level of performance was associated with negative sensations.

« *Once I see the end I can start feeling that all my lips are tired and all my fingers are getting tired* […] *At the end I know that I’m done and I do not have to do it again I just feel I feel lighter* » (M1).

« *The body would not really feel tired but it like my legs are probably getting a little fatigued and to be hitting those times that I was earlier on I’d actually be having to put forth like a much harder effort*» (A2).

Once the high point was reached or the performance was over, the musicians were returned «back in reality» which can be accompanied by feedback from the audience. According to our participants, the end of the performance was associated with relief and relaxation. It was at this point that the musicians regained consciousness of their bodies and felt physical fatigue. Athletes can «come back to reality» in two different ways; either when the performance is over, in which case they would have the same experience as the musicians, or when an alternate event takes place. In the latter case, the exit from the flow state was accompanied by negative emotions.

#### Mental Experience

The experience of leaving the flow state is also lived mentally and is linked with participant affect and focus. Athletes and musicians alike reported mental tiredness resulting in a reduced ability to concentrate. Once again, the emotions felt at the moment depended on the reason why the flow state had been broken. If the reason was due to the performance coming to an end, the emotions that were felt were positive, while the exit from the flow state linked to errors or a decrease in performance level was associated with negative emotions.

« *Yeah I would say constant concentration goes a bit down yes just being able to focus throughout the whole time yes it gets harder* […] » (A5).

« *It’d be more like you know you can lose focus because there’s not as many consequences yeah. And when I guess the flow state stops and all the like the thoughts comeback in and you really relax it’s in a way it’s a breath of fresh air* » (M9).

## Discussion

Several insights regarding the dynamics experienced by athletes and musicians when entering and exiting the flow state can be gleaned from the findings by examining the individual phases (preparation, flow state entry, and flow state exit).

Regarding phase 1 (preparation), which characterized the ways participants entered the flow state, the findings offered an outline of the richness of the views and shared light on a crucial moment where there is a disagreement between researchers. Aherne et al. (2011) considered the flow state in sport to be unpredictable because it cannot be foreseen precisely when, for how long, and how deeply an athlete experiences a flow state. Csikszentmihalyi (1990) argued that the conditions required to enter a flow state are complex but that there are mental approaches that can help lead to flow. Conversely, Jackson (1995) claimed that with proper training, athletes, musicians, and other performers could exercise control over the flow state experience. In the current study, performers highlighted aspects, such as warm-up, focus, body feelings, and emotions, which are in agreement with previous studies that considered flow to be controllable (Jackson, 1995; Sugiyama and Inomata, 2005; Chavez, 2008). Factors, such as physical and mental preparation (Sinnamon, 2020), were highlighted as well as the consideration that nothing is more important than the activity currently being performed, in addition to having the necessary focus on the activity (Biasutti, 2017b). The novel contribution of the current research is the finding that a focus on body feelings and emotions plays a role in flow (Hanin, 2000; Rodgers and Tajet-Foxell, 2011), which could be considered a way of listening to one’s body and capturing inner feelings (Antonini Philippe et al., 2021). This process allows performers to acquire confidence in their skills and performance possibilities (Kirchner, 2011; Swann et al., 2016, 2017; Harris et al., 2017). The physical aspects are particularly relevant for performing activities, such as sports (Jackson and Csikszentmihalyi, 1999) and music (Antonini Philippe et al., 2021). Moreover, emotions are a relevant component for flow (Hanin, 2000; Rodgers and Tajet-Foxell, 2011). For instance, the emphasis on emotions aligns with the emotional drive component identified by Biasutti and Habe (2021) in an interview study on dance flow where participants suggested that true emotions emerged during flow state. In addition, the perceived emotional synchrony with flow seems linked to various social outcomes (Páez et al., 2015), demonstrating the relevance of emotional aspects for flow development.

Regarding phase 2 (entry into the flow states), participants expressed ideas that characterize flow states and how to reach flow. Performers were able to offer examples of their experiences during flow states and identified specific elements, such as goal setting, high level of involvement in the activity, control over the performance, and positive emotions and sensations. These aspects are in line with the nine characteristics of the flow state (Csikszentmihalyi, 1990) with the exception of positive emotions and sensations, which could possibly be a byproduct of being in the flow state. Participants also considered the definition of specific objectives to be important as a way to develop concentration on the activity (Csikszentmihalyi, 1975; Csikszentmihalyi and LeFevre, 1989; Csikszentmihalyi and Nakamura, 2002; Diaz, and Silveira, 2012), thereby enabling a sense of control of the performance (Jackson and Csikszentmihalyi, 1999; Antonini Philippe et al., 2021). In this context, progressive emotions were experienced, and a general feeling of mastery developed (Biasutti and Habe, 2021). The relevance of positive feelings could be verified with physiological measurements of flow, in which muscle activity could be linked to positive emotional experiences accompanying the flow state (Katahira et al., 2018).

Regarding phase 3 (flow state exit), participants, reported several examples of how the flow state gradually fades away. Many elements were identified, such as the end of the performance, physical experience, and mental experience. Participants developed an awareness about the sensations of their experienced flow state and provided evidence regarding the power of the flow state and were able to verbalize how the end of a flow state is experienced. Previous literature (Csikszentmihalyi, 1975, 1990; Csikszentmihalyi and Nakamura, 2002) has focused more on the factors that limit or prevent flow states rather than considering when it disappears.

With respect to the differences between the domains of sport and music, performers reported different attitudes among the phases. In the first phase, the focus of the participants was different: while the musicians’ focus was directed externally, the athletes concentrated their attention internally. This differs however with findings by Singh and Wulf (2020), and suggest that elite athletes are more externally focused. With that said, it is important to note that the type of sport can have an influence. In particular, Singh and Wulf explored flow from the perspective of a team sport, whereas in our study participants all competed in an individual sport. Nonetheless, this finding expands previous research in which no difference was found between athletes and musicians for the factor “total concentration” (Habe et al., 2019). That is, while total concentration could very well be the same, it is possible that the focus of that concentration differs between athletes and musicians, with the former having a stronger external focus, and the latter a stronger internal focus. However, the approaches taken here differ to those of Habe et al. (2019), given the qualitative focus taken here as well as the increased focus on the temporal stages of flow. Conversely, during the second phase that characterized the performance, the focus of both athletes and musicians was directed toward the task. This result is to be expected and in line with previous research on the characteristics of flow state (Csikszentmihalyi, 1975; Csikszentmihalyi and LeFevre, 1989; Csikszentmihalyi and Nakamura, 2002). For the final phase, which involves the end of the flow state and a subsequent return to reality with body and mind experiences, both athletes and musicians report similar feelings. That is, both groups experienced physical fatigue due to effort, positive feelings, and relaxation, assuming that the performance had been successful, or negative feelings if the termination of the flow state was associated with errors or a decrease in the level of performance. These findings reflect a natural behavioral condition in relation to contextual variables.

Although the findings offer several advancements in our conceptualization of flow and how flow is experienced, entered, and left, the study has some noteworthy constraints mainly due to the qualitative nature of our approach. Further, only a relatively few number of athletes and musicians (*n* = 22) participated, making it difficult to generalize these findings. However, our findings offer a detailed understanding of how the flow state is experienced by competitive athletes and elite musicians, providing some input for developing applications of flow state, including educational implications and further research developments.

### Educational Implications and Further Developments

One of the main results of the current study is the role of emotions in regulating flow state (Hanin, 2000; Rodgers and Tajet-Foxell, 2011). Emotions intervene and control flow in all phases for flow, from preparation to termination of flow, in line with previous research (Katahira et al., 2018; Habe et al., 2019). Interestingly, we found that negative emotion is relevant in the experience of flow. Additionally, all participants demonstrated awareness about the power of flow states and provided examples of how to use them for improving sport and music performance. Understanding flow state experiences provides an opportunity to reflect on various levels regarding how they are developed and internalized during sport and musical performance, which could then have pedagogical implications. Indeed, the participants’ description of three distinct phases could be used as a basis for developing educational activities to help advance control over flow states.

There are several additional directions that future research could explore based on the present findings. For example, a deeper analysis of the future aspirations for each group (e.g., a career as a professional musician or athlete). While the participants in our study were highly accomplished and could be reasonably classified as being in the second phase as they transition to experts (see Ericsson et al., 1993), it is possible that flow experiences could differ between (or within) athletes or musicians who have intentions for a professional career when compared with even highly accomplished counterparts. Additionally, it would be important for future research to consider how flow is reported by participants, given that self-reporting flow could be subject to bias or memory confounds. While our procedure took care to first define flow and then use elicitation techniques that presumably facilitate memory, accurately measuring and assessing flow has traditionally been challenging for researchers. Future research could expand on the elicitation interviews used here by increasing the number of questions and allowing the participants additional opportunities to expand on their subjective experiences with the phenomenon of flow. Other aspects to be considered include the differences about how musicians facilitate flow occurrence depending on their motivation in relation to the level of playing being aspired to and whether it is for work or leisure.

Another point for developing further studies is that recent flow research expanded the analysis considering the contributions of the members of the group focusing on what is called group flow, team flow, or shared flow (Pels et al., 2018). Group flow could be relevant for both musicians when performing in chamber groups or in orchestra, and athletes while playing in a team setting. It would be interesting to consider research variables connected to group flow examining if the phases identified in the current research (preparation, flow state entry, and flow state exit) have the same characteristics for group flow.

Considering that the role of the body and physical feelings emerged as playing an important role in flow, further research could analyze the physiological indicators that correspond to specific aspects of the flow experience (Katahira et al., 2018). Regarding emotions, an entire set of investigations is likely to be required to explore the complex emotions and processes involved prior to flow in different domains and in the different levels, motivational orientations, and contexts within even one domain. Additional research should try to integrate all aspects of flow, incorporating behavioral, cognitive, emotional, and neuropsychological characteristics of the flow experience. Lastly, flow has a crucial role in the creative process and is directly linked to optimal experience, and therefore, understanding the underlying mechanisms of flow could be very important for developing mastery and performing optimally.

## Data Availability Statement

The raw data supporting the conclusions of this article will be made available by the authors, without undue reservation.

## Ethics Statement

The studies involving human participants were reviewed and approved by the Committee on Human Subjects at the University of Hawaii at Manoa. The patients/participants provided their written informed consent to participate in this study.

## Author Contributions

SS and RA conceived the study. All authors participated in experimental design and decisions on the experiment specifications. RA, JJ, and SMS collected the data and conducted the interviews. JJ and SMS recruited the participants. RA and MB interpreted the results and drafted the paper. All authors participated in reviewing and revising the manuscript and approved the final version.

## Funding

Open access funding provided by University of Lausanne.

## Conflict of Interest

The authors declare that the research was conducted in the absence of any commercial or financial relationships that could be construed as a potential conflict of interest.

## Publisher’s Note

All claims expressed in this article are solely those of the authors and do not necessarily represent those of their affiliated organizations, or those of the publisher, the editors and the reviewers. Any product that may be evaluated in this article, or claim that may be made by its manufacturer, is not guaranteed or endorsed by the publisher.
