# Supplementary material for: Achieving Flow: An Exploratory Investigation of Elite College Athletes and Musicians
Source: Front Psychol. 2022 Mar 30;13:831508. doi: 10.3389/fpsyg.2022.831508 (PMC9009586; doi:10.3389/fpsyg.2022.831508)
Supplement: Supplementary file 1 [file Data_Sheet_1.PDF]

Supplementary Material

1.1 Supplementary Figures

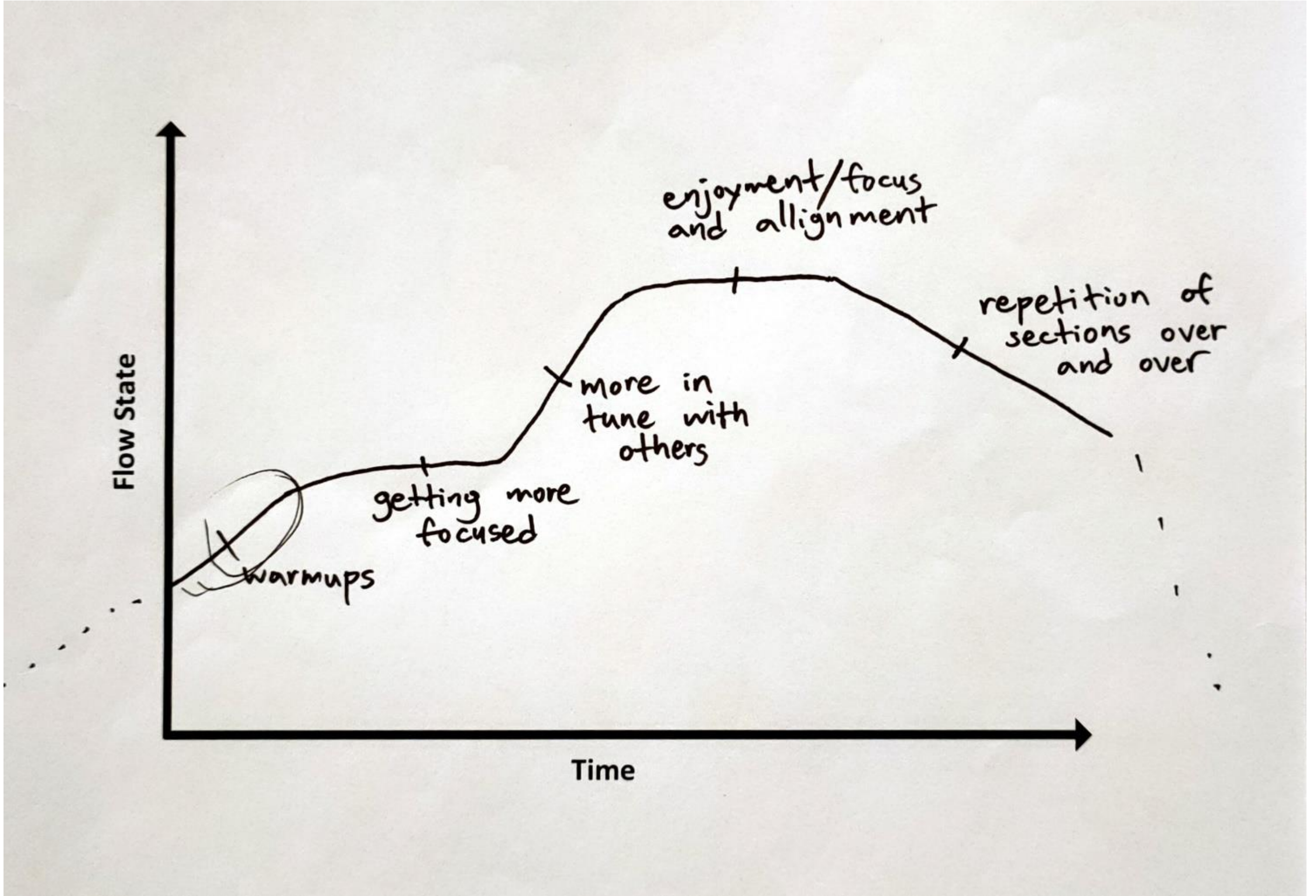

Supplementary Figure 1. Self-perceived intensity of the flow state in relation to the temporal progression: Exemplary visualization by a participant of the study.
